# A solitary fibrous tumor in the pelvic cavity of a patient with Doege-Potter syndrome: a case report

**DOI:** 10.1186/s40792-019-0617-6

**Published:** 2019-04-11

**Authors:** Yukiko Wada, Keiichi Okano, Yasuhisa Ando, Jun Uemura, Hironobu Suto, Eisuke Asano, Takayoshi Kishino, Minoru Oshima, Kensuke Kumamoto, Hisashi Usuki, Yasuyuki Suzuki

**Affiliations:** Department of Gastroenterological Surgery, Kagawa University, 1750-1 Ikenobe, Miki-cho, Kita-gun, Kagawa 761-0793 Japan

**Keywords:** Solitary fibrous tumor, Hypoglycemia, Pelvic

## Abstract

**Background:**

A solitary fibrous tumor (SFT) is a mesenchymal lesion, which commonly develops in the thorax. Non-islet cell hypoglycemia is a rare paraneoplastic phenomenon caused by an extra-pancreatic tumor. We report a rare case of a pelvic SFT with severe hypoglycemia, which was considered to be Doege-Potter syndrome.

**Case presentation:**

A 72-year-old man was referred to our hospital for treatment of hypoglycemia and a large pelvic tumor. His blood glucose level was 52 mg/dl; serum insulin level, 1.0 μIU/ml; C-peptide level, 0.2 ng/ml; and insulin-like growth factor-I (IGF-I) level, 31 ng/ml. Contrast-enhanced computed tomography (CT) showed a 13-cm mass in the pelvic cavity. Magnetic resonance imaging (MRI) revealed a lobulated tumor with iso- and high-intensity areas combined in T2-weighted images. No clear invasion to any adjacent organs was identified. The tumor was resected, and hypoglycemic symptoms disappeared immediately. Pathological diagnosis was an SFT with malignant potential that secreted IGF-II and caused hypoglycemia. There has been no tumor recurrence during the 1 year of follow-up.

**Conclusion:**

Non-islet cell tumor hypoglycemia should be considered in the differential diagnosis of patients presenting with tumors and hypoglycemia.

## Background

Solitary fibrous tumor (SFT) was first reported by Klemperer and Rabin in 1931 as a lesion that originated in the pleura [1]. However, SFTs are now mesenchymal tumors and develop in various anatomic locations. Approximately 80% of SFTs are located in the thoracic cavity [2]. Among extra-thoracic SFTs, the occurrence of a primary SFT in the pelvic cavity is rare, with a reported incidence of 16% among extra-thoracic SFTs [3, 4]. The incidence of SFT is 2.8 per 100,000 people [5], the age onset of SFT is around 50–60 years, and the incidence among males and females is nearly equal [6]. Non-islet cell tumor hypoglycemia (NICTH) is a rare paraneoplastic condition caused by an extra-pancreatic tumor. NICTH is observed in only 4% of thoracic SFTs, and this condition is referred to as Doege-Potter syndrome [4, 7]. Hypoglycemia develops due to the production of insulin-like growth factor (IGF)II by the tumor. We report a case of pelvic SFT with Doege-Potter syndrome.

## Case presentation

A 72-year-old man was admitted to the emergency department for a hypoglycemic attack. The computed tomography (CT) scan detected a large tumor in the pelvic cavity, and he was referred to our hospital for closer examination. The patient had no relevant medical history and was not on any medication. On admission, his blood glucose level was 52 mg/dl (normal range 70–109 mg/dl); serum insulin level, 1.0 μIU/ml (normal range 3–15 μIU/ml); C-peptide level, 0.2 ng/ml (normal range 0.43–2.35 ng/ml), and IGF-I level, 31 ng/ml (normal range 58–198 ng/ml).

**Fig. 1:**
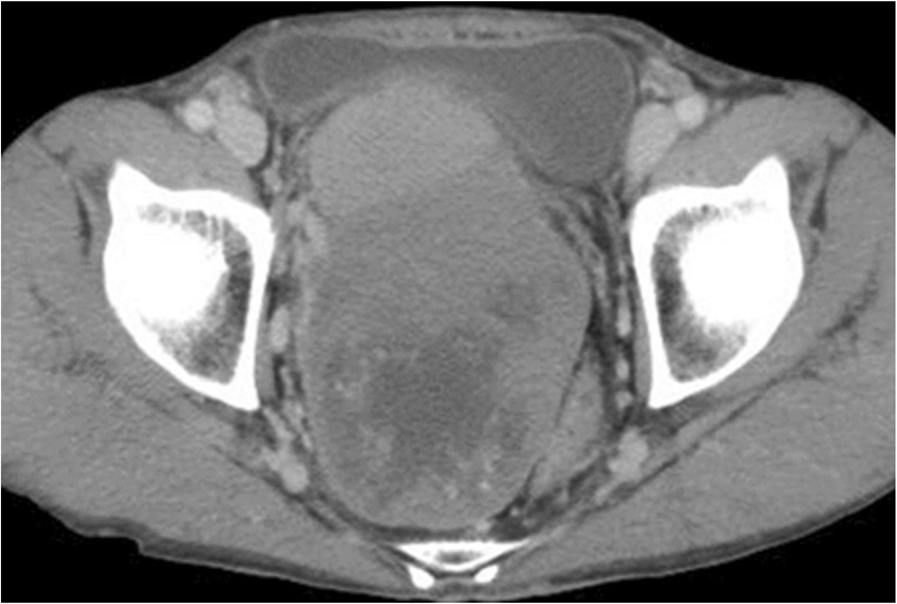
Contrast-enhanced CT image showing a mass occupying the pelvic cavity. The tumor was heterogeneously enhanced

**Fig. 2:**
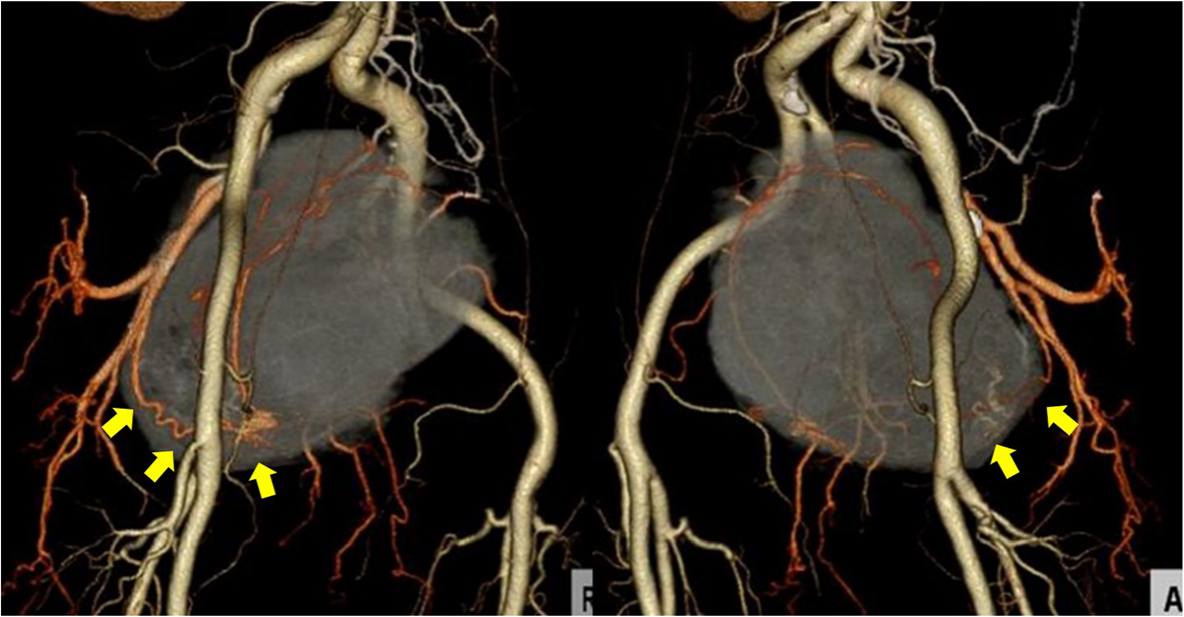
CT-angiography image showing the feeding vessels of the tumor branching from the right and left internal iliac arteries (arrow)

**Fig. 3:**
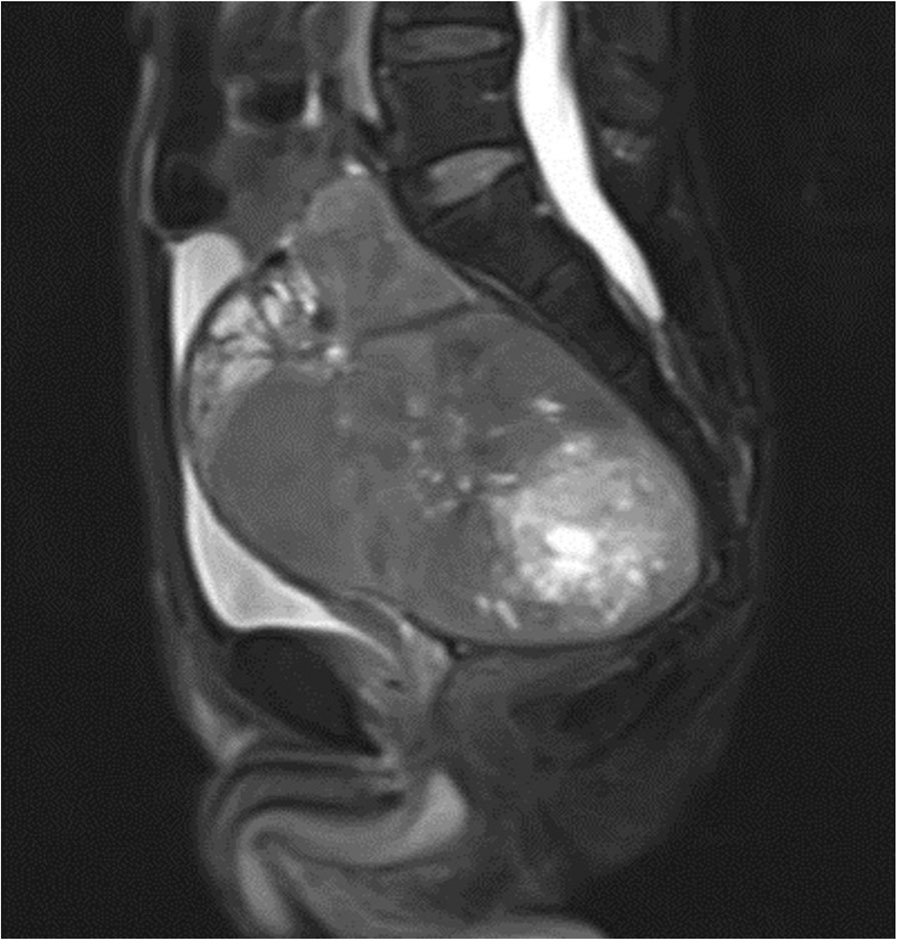
T2-weighted pelvic MRI image. No clear invasion to any adjacent organs is identified

**Fig. 4:**
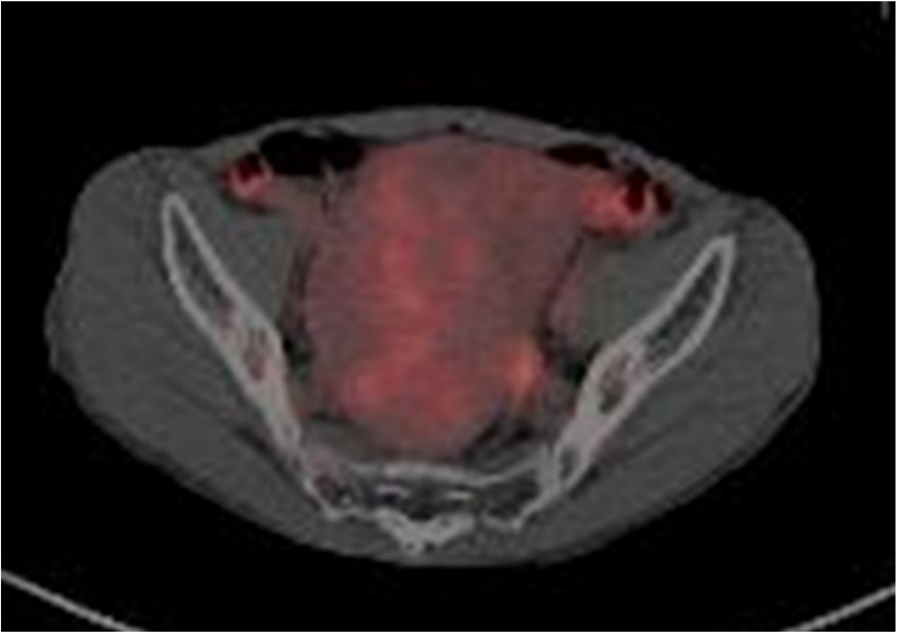
PET-CT image showing heterogeneous accumulation on the tumor. SUV-max level is 2.5

Contrast-enhanced CT indicated a heterogeneous spheroid mass with little contrast-enhancement measuring 13 × 9 × 11 cm in the pelvic cavity (Fig. 1). CT-angiography revealed the presence of feeding vessels branching from the right and left internal iliac arteries (Fig. 2). Magnetic resonance imaging (MRI) revealed a lobulated tumor with iso- and low-intensity areas combined in T1-weighted images, and iso- and high-intensity areas combined in T2-weighted images. No clear invasion to any adjacent organs was identified (Fig. 3). Positron emission tomography (PET)-CT revealed heterogeneous accumulation on the tumor with a maximum standardized uptake value (SUVmax) of 2.5 (Fig. 4).

Hypoglycemia was observed despite continuous glucose infusion, and glucocorticoid administration was initiated prior to tumor resection. The tumor was located retroperitoneally, fed by the superior vesical artery, and was completely excised. Operation time was 388 min, and intraoperative blood loss was 1410 ml.

**Fig. 5:**
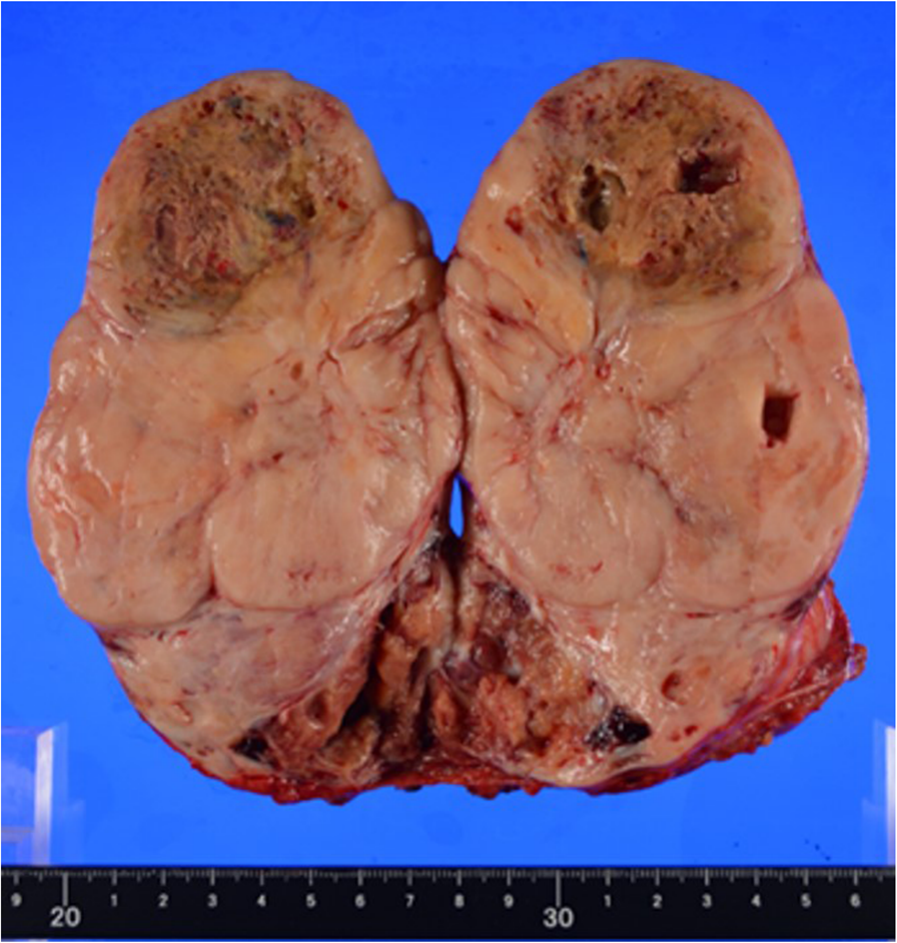
Macroscopic view of the resected tumor. The 15 × 8 × 8-cm tumor has a fibrous capsule and is composed of partially necrotic grayish-white tissues

**Fig. 6:**
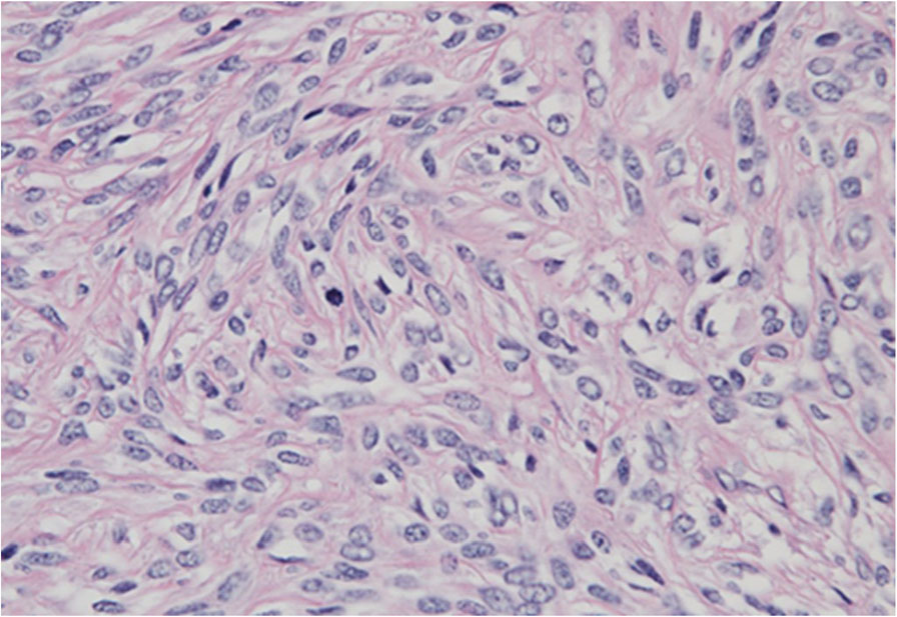
Histological examination (HE stain:×tai magnification) shows patternless architecture involving spindle cells

**Fig. 7:**
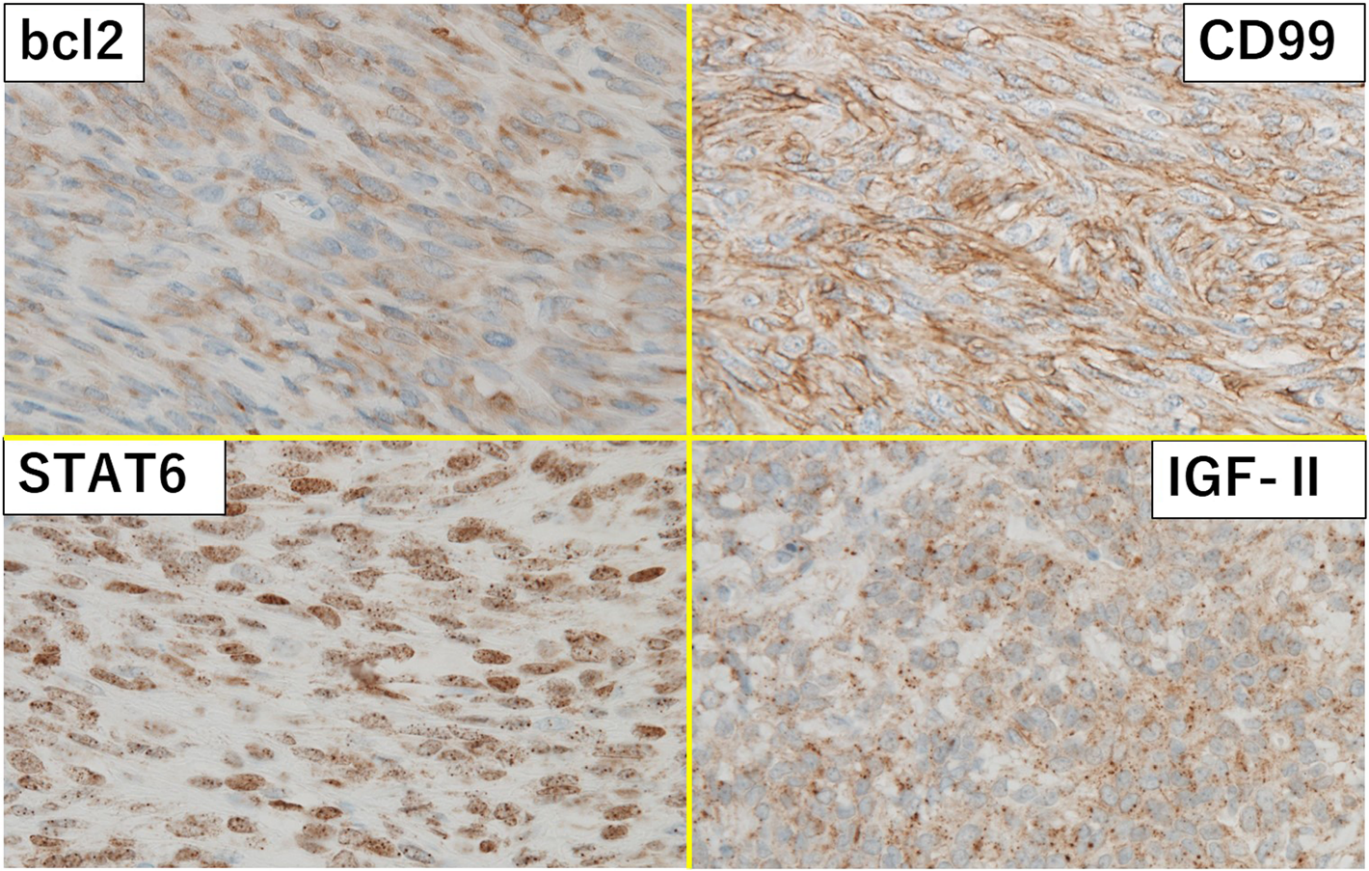
Immunohistochemical staining showing positive staining for STAT6, CD99, bcl-2, and insulin-like growth factor-II (IGF-II)

Macroscopically, the tumor was solid and composed of partially necrotic grayish-white tissue; the tumor measured 15 × 8 × 8 cm (Fig. 5) and had a fibrous capsule. When observed under the microscope, the tumor was composed of spindle cells arranged in no particular pattern (Fig. 6). Seven mitoses were counted per 10 high-power fields (HPF). Immunohistochemical staining revealed that the tumor was positive for signal transducers and activators of transcription 6 (STAT6), CD99, bcl-2, insulin-like growth factor-2 (IGF-II), and CD34, and negative for CD31, EMA, S-100, SMA, and desmin. From these findings, we diagnosed this tumor as an SFT with malignant potential that secreted IGF II, which caused hypoglycemia (Fig. 7).

The postoperative course was uneventful, and hypoglycemic symptoms disappeared immediately after surgery. There has been no tumor recurrence during the 1 year of follow-up.

## Discussion

SFTs are typically benign, but they can be malignant, particularly if they become large or in cases of repeated recurrence. Approximately 12–22% of SFTs are found to be malignant [6]. In NICTH cases, however, the frequency of malignancy was 60% [4]. This suggested that SFTs with NICTH were more likely to be malignant. NICTH is observed in 4% of thoracic SFTs, and only nine cases of pelvic SFT with hypoglycemia have been reported in the literature [8–16].

The 2013 WHO classification of soft tissue tumors defines malignancy as hypercellular, mitotically active (> 4 mitoses/10 HPF) tumors with cytological atypia, tumor necrosis, and/or infiltrative margins [17]. The present case was diagnosed as malignant because of a high mitosis rate and tumor necrosis.

In 2013, recurrent nerve growth factor-inducible A binding protein-2 (NAB2) and signal transducers and activators of transcription-6 (STAT6) gene fusion were identified in SFT by whole exome sequencing [18]. A strong nuclear STAT6 signal was detected immunohistochemically as a result of the presence of the NAB2-STAT6 fusion gene, which could be helpful in diagnosing SFT. In addition, CD34, Bcl-2, vimentin, and S100 expression were often observed in SFT [17].

NICTH is a rare paraneoplastic phenomenon found mainly in large mesenchymal tumors such as SFT, fibrosarcoma, mesothelioma, gastrointestinal stromal tumor, and hemangiopercytoma [19]. Doege and Potter first described NICTH in patients with SFT, and few systemic studies have been published about this condition because of its rare incidence. NICTH-causing tumors produce and release IGF-II into circulation, which has a high affinity for the insulin receptor. Normally, IGF-II forms a trimer by binding to IGF-binding proteins; thus the trimer does not pass from the circulation to the extracellular space. In NICTH, the tumor produces high molecular weight IGF-II, which has a low affinity for IGF binding proteins. As a high molecular weight IGF-II cannot form trimer, it exists in a free form in serum. Free IGF-II easily pass through capillaries and it reach the insulin receptor in the target cell [20]. In this way, high molecular weight IGF-II witch the tumor produce causes hypoglycemia. Serum levels of IGF-II are either normal or elevated in the standard detection method, and quantification of tumor-related high molecular weight IGF-II levels is difficult. Immunoblot analysis of IGF-II is more accurate for establishing diagnosis, but these procedures often are not feasible in routine clinical settings. In clinical practice, the possibility of NICTH secondary to IGF-II is suggested by low blood glucose, with suppressed levels of serum insulin, IGF-I, C-peptide, and growth hormone [19, 21].

Complete tumor resection was reported to be the definitive treatment of SFT. Radiotherapy or chemotherapy is often applied to unresectable or metastatic tumors. However, SFTs are considered relatively chemoresistant, and there are no standard chemotherapeutic regimens. Resectability remains the most important prognostic factor [4]. Preoperative percutaneous embolization was reported to reduce tumor volume and intraoperative blood loss, but the effect is not confirmed [22].

## Conclusion

We reported a rare case of NICTH of SFT in the pelvic cavity. When a tumor with hypoglycemia is detected, the possibility of NICTH should be considered.

## Abbreviations

CT: Computed tomography
HPF: High-power field
IGF: Insulin-like growth factor
MRI: Magnetic resonance imaging
NAB2: Nerve growth factor-inducible A binding protein 2
NICTH: Non-islet cell tumor hypoglycemia
PET: Positron emission tomography
SFT: Solitary fibrous tumor
STAT6: Signal transducers and activators of transcription 6
